# Production of Marinated Chinese Lotus Root Slices Using High-Pressure Processing as an Alternative to Traditional Thermal-and-Soaking Procedure

**DOI:** 10.3390/molecules27196506

**Published:** 2022-10-02

**Authors:** Lin Yuan, Feifei Xu, Yingying Xu, Jihong Wu, Fei Lao

**Affiliations:** 1 College of Food Science and Nutritional Engineering, China Agricultural University, Beijing 100083, China; 2 National Engineering Research Center for Fruit and Vegetable Processing, Beijing 100083, China; 3 Key Laboratory of Fruit and Vegetable Processing, Ministry of Agriculture and Rural Affairs, Beijing 100083, China; 4 Beijing Key Laboratory for Food Non-Thermal Processing, Beijing 100083, China; 5 Xinghua Industrial Research Centre for Food Science and Human Health, China Agricultural University, Xinghua 225700, China

**Keywords:** flavor, color, texture, total viable counts, shelf life, Arrhenius equation

## Abstract

Marinated vegetables are traditional cold dishes with a long history and special flavor in the Chinese deli market. However, the traditional thermal-and-soaking (TS) procedure often results in unreproducible flavor quality properties of marinated vegetables and waste of brine and time in production. A novel green and sustainable technique, high-pressure processing (HPP), has caught the attention of the food industry. In this study, the effects of HPP and TS treatment on the visual, flavor, textural, and microbiological qualities of Chinese marinated lotus root slices were investigated. Compared to the TS products, lighter color, more varieties of volatile compounds, and crunchier texture were detected in the HPP products. Throughout the 4 °C, 25 °C, and 45 °C shelf life challenges, the HPP products retained their original color and crunchiness better than the TS ones, whereas no significant differences were found in total viable counts (TVCs) in the first half of the shelf lives. The Arrhenius model under the first-order reaction of TVC deterioration showed a good fit to the shelf life of the HPP marinated lotus root slices. This study demonstrates that HPP may assist in making the best use of brine in a more time-efficient manner to improve the visual, flavor, and textural quality of traditional Chinese marinated lotus root slices.

## 1. Introduction

Marinated vegetables are popular in the Chinese deli market; they are traditional cold dishes with a history that can be traced back to the Qin dynasty (BC221). Unlike food cooked using other methods, marinated vegetables exhibit a delightfully crunchy texture and unique flavor and are abundant in nutrients [1,2,3,4]. The most commonly marinated vegetables are lotus root, potato, asparagus, bamboo shoot, and bean curd products. Traditionally, marinated vegetables are boiled for a period of time in brine containing seasonings and spices, such as salt, soy sauce, vinegar, garlic, pepper, and ginger, and then soaked overnight before serving [5]. This dish is conventionally consumed immediately after preparation or after short-term storage under refrigerated conditions. Recently, ready-to-eat marinated vegetables with extended shelf lives have bloomed in the snack market. However, finding a balance between saving brine utilization and stabilizing product flavor and texture quality has been a challenge faced by most manufacturers. Moreover, the industry urgently needs to upgrade the long boiling-and-soaking production cycle, targeting higher time and energy efficiency.

A novel green and sustainable non-thermal sterilization technique, high-pressure processing (HPP), has caught the attention of the food industry in the past decades and has been reported to be effective in saving water and energy [6,7]. Currently, the application of HPP has been innovatively expanded into areas such as food pretreatment, extraction facilitation, enzyme activity control, and food flavor improvement [8,9,10,11]. In marinated foods, HPP has been used mainly for shelf life extension and quality maintenance. Bao et al. [1] found that treatment at 550 MPa for 5 min can effectively inactivate microorganisms in fermented marinated radish with less microstructural damage and better flavor compared to thermal processing. Rodrigues et al. [12] evaluated the impact of HPP on the microbiological properties of marinated beef after refrigerated storage for 14 days and found that HPP led to a reduction of 6 log_10_ colony-forming units (CFU)/g in the two tested microorganisms. Scheinberg et al. [13] reported similar results for bacterial reduction in beef jerky. Moreover, O’Neill et al. [14] reported that HPP at 300, 400, and 500 MPa extended the shelf life of marinated pork chops by 16, 22, and 29 days, respectively. Since the prolongation of shelf life by HPP is mainly due to the inhibition of microbial growth and enzymatic activity [15], the determination of post-HPP food quality changes in storage and prediction of food shelf life based on microbial or enzymatic status is important and would have great practical value.

Several recent studies have developed mathematical models based on quality changes to predict and optimize the shelf life of HPP-treated foods. For example, the polynomial regression model and dimensionless nonlinear model were used to predict the inactivation of *Salmonella* under different HPP conditions in raw ground chicken meat [16]. The shelf life of human milk treated by HPP, ultraviolet light, and pasteurization was determined at 25 °C and 40 °C using the Arrhenius model based on the percentage of free fatty acids [17]. However, these studies have focused on HPP-treated animal products; similar models for HPP-treated marinated vegetable products are needed as well.

The lotus root (*Nelumbo nucifera* Gaertn.) belongs to the Nymphaeaceae family and is an aquatic vegetable containing alkaloids, flavonoids, niacin, vitamins, and other bioactive components, thus possessing a high nutritional and medicinal value [18]. The marinated lotus root is a traditional Chinese unfermented ready-to-eat food with high industrial potential. Marinated lotus root slices processed with the traditional thermal-and-soaking (TS) method require a large amount of brine and time; a more advanced green and efficient processing method is in high demand. Hee et al. [19] evaluated the effects of adding different concentrations of beet water extract during storage on the quality characteristics of marinated lotus roots. Li et al. [20] investigated the effects of pulsed electric field pretreatment on the mass transfer kinetics of lotus root slices during marination. Yet, these studies emphasizing novel processing technologies have focused on the safety and taste quality of marinated foods, with little attention paid to volatile aroma substances that directly affect consumer preferences. Investigating the effect of the processing method on the aroma profile of marinated lotus root slices would provide a more comprehensive understanding of the sensory properties of the product.

Therefore, this study aimed to investigate the feasibility of using HPP for the efficient processing of Chinese marinated lotus root slices. The effects of HPP and TS treatment on the flavor, visual, textural, and microbiological qualities of Chinese marinated lotus root slices were investigated. A shelf life model was created to predict the lotus root product performance in different storage temperatures based on the product quality data collected. The information in this study is an important reference to traditional food production modernization, aiming for higher processing efficiency and potential quality improvement.

## 2. Results and Discussion

### 2.1. Effect of HPP and TS Processing on the Quality of Marinated Lotus Root Slices

The control sample was excluded from the following experiments because it had a “plastic odor” in the previous sensory evaluation, probably due to the unpleasant aroma release caused by heating the samples packaged with plastic materials [21].

#### 2.1.1. Volatile Compounds

Aroma is an important aspect of the quality evaluation of marinated lotus root slices. The volatile compounds in the blank and marinated lotus root slices are listed in Table 1. In total, 18 volatile compounds were identified in the blank marinated lotus root slices, whereas 32 and 26 volatile compounds were identified in the HPP- and TS-processed marinated lotus root slices, respectively. The blank lotus root has a bland and neutral aroma, and the main volatile compounds such as alkyls in the blank lotus root are contributed by the soil and water environments [22]. Most of the volatile compounds in the marinated lotus root slices detected in this study were from the spices, herbs, and seasonings in the brine. Higher concentrations of total volatile compounds were detected in the TS samples than in the HPP samples but not significantly. Phenols (e.g., eugenol), heterocyclic compounds (e.g., ethyl maltol), alcohols (e.g., benzyl alcohol), ethers (e.g., *cis*-anethol), and aldehydes (e.g., benzaldehyde) were the main volatile categories in marinated lotus root slices. These compounds were previously identified as the major contributors to the aromatic components of spices such as clove and fennel [23,24,25,26]. Although all other categories of volatile compounds were significantly (*p <* 0.05) higher in the TS samples than in the HPP samples, the volatile phenols with the highest content were not significantly different (*p >* 0.05) between the TS and HPP samples. This suggests that HPP can promote the release of volatile compounds at a small amount of brine use to achieve similar aroma quality as TS treatment.

#### 2.1.2. Color, Texture, and TVC

Table 2 shows the color, texture, and microbiological properties of HPP and TS samples. In contrast to the TS treatment, HPP increased the hardness value of marinated lotus root slices. Basak and Ramaswamy [27] defined instantaneous pressure softening (IPS), i.e., the loss in texture that occurs at the instant of HPP (zero holding time), by measuring and fitting the hardness of HPP-treated fruits and vegetables treated at 100–400 MPa for 5–60 min. The IPS will be recovered to varying degrees with increasing pressurization time. The HPP conditions at 550 MPa with a holding time of 20 min in this study seem to have accomplished this textural recovery. In addition, as reported in previous studies, HPP also increases the hardness of some other marinated products, such as marinated fermented radish [1], marinated beef [11], and calcium-infused baby carrots [28]. This may be due to the aggregation of proteins/peptides [29] or marination-induced changes in cell microstructure, such as membrane permeability, cellular rearrangement, and cell collapse [1,28,30], which alter the effect of HPP on cell volume compression.

The TVC in samples before HPP or TS was 3.98 log_10_ CFU/g and immediately reduced to 1.68 and 1.60 log_10_ CFU/g after HPP and TS treatments, respectively. The main mechanism of action of HPP in microorganisms involves altering cell structure and physiological functions, breaking DNA strands, disrupting cell membrane integrity, inactivating key enzymes, and irreversible denaturation of proteins, resulting in the loss of membrane selectivity [31]. It can be inferred that the bacteria with tolerance to the thermal process may be the thermal-tolerance spores, such as Bacillus and Clostridium [32]. Additionally, the TVC result of HPP samples was not significantly (*p* > 0.05) different from that of TS samples. Therefore, it was speculated that the survivor bacteria after HPP may be the spores because the spores have a high tolerance to HPP [33]. However, according to the shelf-life experiments later in this study, these spores did not have a destructive effect on the quality of marinated lotus root slices.

The pH of the samples decreased significantly (*p* < 0.05) after HPP or TS treatment, and the pH of the HPP samples was slightly but significantly (*p* < 0.05) higher than that of the TS samples, which may be due to the fact that HPP facilitates acidic substance transfer to less of an extent than TS treatment. However, the pH variation of 0.09 is a considerably small change in batch marinated food processing and would not introduce perceivable acidity alteration.

**Figure 1:**
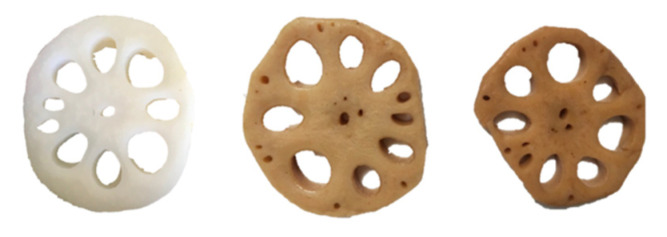
The appearance of blank (**left**), HPP (**middle**), and TS (**right**) marinated lotus root slices. HPP, high-pressure processing; TS, thermal-and-soaking.

It was observed that HPP and TS processing resulted in a noticeable visual difference in color (Figure 1). Immediately after HPP and TS processing, the L* value of marinated lotus root slices significantly decreased, whereas the a* and b* values increased. The ΔE value of the samples after TS processing was 36.41, which was significantly higher than that of the HPP samples (ΔE = 32.20). This indicates a darker brightness and a redder and more yellowish color of the TS samples compared to the HPP samples. These color changes may be related to cell membrane disruption, thermal denaturation, and the Maillard reaction [34,35].

### 2.2. Effects of HPP and TS Processing on Quality Properties during Different Storage Conditions

#### 2.2.1. Texture Changes

**Figure 2:**
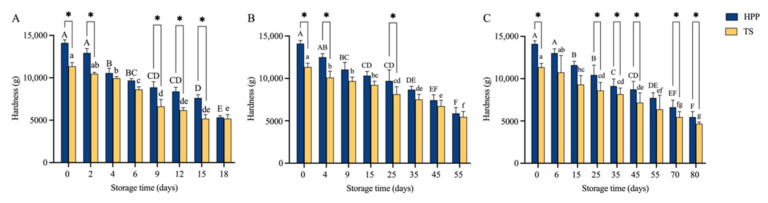
Changes in hardness of HPP and TS marinated lotus root slices during storage: (**A**) storage at 45 °C, (**B**) storage at 25 °C, (**C**) storage at 4 °C. Different letters indicate significant differences during storage (*p* < 0.05) for the HPP (uppercase letters) and TS (lowercase letters) marinated lotus root slices. * *p* < 0.05. HPP, high-pressure processing; TS, thermal-and-soaking.

Softening of fruits and vegetables is a process of destruction of the cell structure caused by the breaking of cell walls and dissolution of pectin by the actions of enzymes. During storage, cell rupture due to temperature changes, microbial growth, and a series of biochemical reactions eventually lead to a decrease in product hardness. Therefore, the hardness of fruits and vegetables is often used as an important indicator of quality changes during storage [36,37]. As shown in Figure 2, the hardness of marinated lotus root slices tended to decrease at the three temperatures during storage and finally reached a value of approximately 5000 g at the end of the shelf life. The hardness values of the marinated lotus root slices decreased faster at higher storage temperatures. These findings were consistent with those of previous studies on atemoya [38]. The most important softening process in the HPP or TS process is considered to be the temperature-dependent β-elimination of pectin [39]. In addition, most of the hardness values of HPP marinated lotus root slices were significantly higher (*p <* 0.05) than those of TS marinated lotus root slices stored at the same temperature (Figure 2). This finding was consistent with the study by Zhang et al. [40] on yellow peaches in pouches, indicating that HPP could maintain the texture of food. This is due to the release of PME under HPP treatment, which catalyzed the demethylation of high-methylated pectin, and the resulting de-esterified pectin (low-methylated pectin) forms a gel network with divalent ions [41].

#### 2.2.2. Color Changes

Table S1 and Table 3 show the changes in L*, a*, b*, and ΔE values in the HPP and TS marinated lotus root slices during storage at 45 °C, 25 °C, and 4 °C. The quality of the lotus root is mainly reflected by the changes in color, specifically in lightness and yellowness [42]. The L* values of both HPP and TS marinated lotus root slices decreased continuously with storage time, indicating that the color darkened during storage. There was a significant decrease in b* values for marinated lotus root slices during storage, indicating that the yellow hue in marinated lotus root slices degraded significantly. This result was similar to that of a previous study on HPP carrots and thermally processed strawberry puree [30,43]. The ΔE values of marinated lotus root slices significantly increased during storage, indicating that the color quality declined as the storage period increased. The color changes observed in marinated lotus roots during storage may be due to a variety of causes, such as enzymatic or non-enzymatic browning reactions and microbial spoilage [44,45]. However, the ΔE values of the HPP samples were consistently lower than those of the TS samples of the same period at the same storage temperature. Similar results were also found for marinated radish and cloudy kiwifruit juice [1,46]. Therefore, HPP can maintain the original color of the marinated lotus root slices better than the TS process.

In addition, the color of marinated lotus root slices was also affected by storage temperature. HPP and TS samples stored at 4 °C showed more stable color quality than samples stored at 25 °C, with the worst quality being observed in samples stored at 45 °C. The rate of increase in the ΔE values increased with increasing storage temperature, indicating that the rate of browning reactions was accelerated at high storage temperatures. Min et al. [42] reported similar results for freshly cut lotus root. This is because low temperatures reduce enzymatic browning reactions by decreasing enzyme activity and also reduce non-enzymatic browning reactions such as the Maillard reactions and ascorbic acid degradation [35,42,47]. Based on previously published reports, it is unclear whether the effect of storage temperature on the color of HPP samples differs from that of thermally processed samples. Studies performed on orange juice [48] and aloe vera–litchi mixed beverage [49] showed that the color change of HPP samples was more affected by storage temperature than that of thermally processed samples. Opposite results have been reported for clear and cloudy Se-enriched kiwifruit juices [45]. In our study, the color changes of HPP and TS marinated lotus root slices were similarly affected by storage temperature. Therefore, appropriate storage temperatures combined with optimal processing technology help to better preserve the appearance of marinated lotus root slices.

#### 2.2.3. TVC Changes

**Figure 3:**
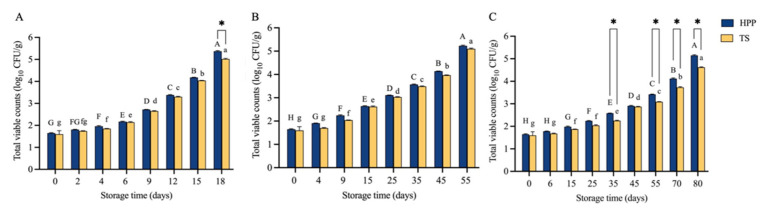
Changes in total viable counts of HPP and TS marinated lotus root slices during storage: (**A**) storage at 45 °C, (**B**) storage at 25 °C, (**C**) storage at 4 °C. Different letters indicate significant differences during storage (*p* < 0.05) for the HPP (uppercase letters) and TS (lowercase letters) marinated lotus root slices. * *p* < 0.05. HPP, high-pressure processing; TS, thermal-and-soaking.

TVC in HPP and TS marinated lotus root slices stored at different temperatures increased at different rates, reaching 5 log_10_ CFU/g at 80, 55, and 18 days of storage at 4, 25, and 45 °C, respectively (Figure 3). The sample microbial growth was the fastest when stored at 45 °C and slowest at 4 °C. Except for samples stored at 45 °C on day 18 and at 4 °C on days 35, 55, 70, and 80, there was no significant difference in TVC between HPP and TS marinated lotus root slices (*p* > 0.05), indicating that HPP could achieve the same sterilization effect as conventional TS processing in the first half of the shelf lives. Similar results have been reported regarding many fruit and vegetable products [50,51], and HPP is microbiologically safe and the best alternative to thermal processing [52]. However, some studies have also shown that the sterilization effect of HPP is superior [53] or inferior [54] to that of thermal processing, which may be related to pressure, temperature, time, and pH [52].

### 2.3. Shelf Life Prediction Models of HPP Marinated Lotus Root Slices

Zero- and first-order kinetic reaction equations (Equations (1) and (2)) are commonly used to construct food quality reaction kinetic models [55].

(1) $$\begin{array}{c} \mathrm{Zero}-\mathrm{order}\;\mathrm{reaction}\;\mathrm{model}:C=\mathrm{kt}+C_{0} \end{array}$$

(2) $$\begin{array}{c} \mathrm{First}-\mathrm{order}\;\mathrm{reation}\;\mathrm{model}:C=C_{0}e^{\mathrm{kt}} \end{array}$$

where t is the storage time (days), C is the index value of storage time t, C_0_ is the initial value of the index, and k is the decay rate of the index.

The hardness, TVC, and ΔE data of HPP marinated lotus root slices stored at different storage temperatures and times in Figure 2 and Figure 3 and Table 3 were fitted with Equation (1) or (2). The corresponding zero- and first-order reaction rate constants (k) and the determination coefficients (R^2^) are listed in Table S2. The average R^2^ values of the kinetic equations for changes in hardness and TVC under the first-order reaction (0.9688 and 0.9900, respectively) were slightly higher than those of the zero-order reaction (0.9524 and 0.9571, respectively), whereas the average R^2^ values of the zero- and first-order reactions for ΔE changes were similar (0.9000 and 0.9029, respectively). It has been reported that the changes in the hardness of white button mushrooms and the TVC of grass carp follow the first-order reaction, and the change in ΔE of tambaqui fillet follows the zero-order reaction [56,57,58].

A quantitative description of the relationship between the temperature and the rate of a chemical reaction was further performed using the Arrhenius equation (Equation (3)) [55].

(3) $$\begin{array}{c} \mathrm{lnk}=\ln k_{0}-\frac{\mathrm{Ea}}{\mathrm{RT}} \end{array}$$

where k is the deterioration rate, k_0_ is the pre-exponential factor, Ea is the activation energy (J/mol), T is the storage temperature (K), and R is the universal gas constant (8.314 J/(mol·K)).

According to Equation (3), the reaction rate constant (k) for hardness, TVC, and ΔE of the HPP marinated lotus root slices in Table S2 were taken logarithmically and linearly fitted to the corresponding 1/T. Ea and k_0_ were calculated from the slope and intercept of the fitted equations, respectively, as shown in Table 4.

By combining the corresponding zero- or first-order kinetic model (Equation (1) or (2)) with the Arrhenius equation (Equation (3)), shelf life prediction models with the variables of temperature (T) and quality factor (C) were established, as shown in Equations (4) and (5):

The Arrhenius model under the zero-order reaction:

(4) $$\begin{array}{c} \mathrm{SL}=\frac{\left|C-C_{0}\right|}{k_{0}e^{-\frac{\mathrm{Ea}}{\mathrm{RT}}}} \end{array}$$

The Arrhenius model under the first-order reaction:

(5) $$\begin{array}{c} \mathrm{SL}=\frac{\left|\ln\frac{C}{C_{0}}\right|}{k_{0}e^{-\frac{\mathrm{Ea}}{\mathrm{RT}}}} \end{array}$$

where SL is the shelf life (days) of HPP marinated lotus root slices, C is the index value of storage time T, C_0_ is the initial value of the index, k_0_ is the pre-exponential factor, Ea is the activation energy (J/mol), T is the storage temperature (K), and R is the universal gas constant (8.314 J/(mol·K)).

These models could be used to assess the quality of HPP marinated lotus root slices after storage at a given temperature and storage duration, as well as to acquire the real storage time corresponding to a certain quality value.

The k_0_ and Ea corresponding to hardness, TVC, and ΔE under zero- or first-order reactions in Table 4 were substituted into Equation (4) or (5), respectively, to obtain the shelf life prediction model (models under zero-order reaction are shown as Equations (6a) to (8a), models under first-order reaction are shown as Equations (6b) to (8b)).

The shelf life prediction models of hardness:

(6a) $$\begin{array}{c} {\mathrm{SL}}_{\mathrm{hardness},\mathrm{zero}}=\frac{\left|C_{\mathrm{hardness}}-C_{\mathrm{hardness}0}\right|}{4497355\times e^{-\frac{3007}{T}}} \end{array}$$

(6b) $$\begin{array}{c} {\mathrm{SL}}_{\mathrm{hardness},\mathrm{first}}=\frac{\left|\ln\frac{C_{\mathrm{hardness}}}{C_{\mathrm{hardness}0}}\right|}{556.7\times e^{-\frac{3046.6}{T}}} \end{array}$$

The shelf life prediction models of TVC:

(7a) $$\begin{array}{c} {\mathrm{SL}}_{\mathrm{TVC},\;\mathrm{zero}}=\frac{\left|C_{\mathrm{TVC}}-C_{\mathrm{TVC}0}\right|}{6494\times e^{-\frac{3358.7}{T}}} \end{array}$$

(7b) $$\begin{array}{c} {\mathrm{SL}}_{\mathrm{TVC},\;\mathrm{first}}=\frac{\left|\ln\frac{C_{\mathrm{TVC}}}{C_{\mathrm{TVC}0}}\right|}{1790\times e^{-\frac{3303.9}{T}}} \end{array}$$

The shelf life prediction models of ΔE:

(8a) $$\begin{array}{c} {\mathrm{SL}}_{\Delta E,\mathrm{zero}}=\frac{\left|C_{\Delta E}-C_{\Delta E0}\right|}{1313\times e^{-\frac{2699.3}{T}}} \end{array}$$

(8b) $$\begin{array}{c} {\mathrm{SL}}_{\Delta E,\mathrm{first}}=\frac{\left|\ln\frac{C_{\Delta E}}{C_{\Delta E0}}\right|}{32.54\times e^{-\frac{2664.3}{T}}} \end{array}$$

where SL is the predicted shelf life (days) of HPP marinated lotus root slices; T is the storage temperature (K); C_hardness_, C_TVC_, and C_ΔE_ are predicted values of hardness, TVC, and ΔE of HPP marinated lotus root slices after storage for time SL; and C_hardness0_, C_TVC0_, and C_ΔE0_ are initial values of hardness, TVC, and ΔE of HPP marinated lotus root slices.

To evaluate the shelf life prediction models of hardness, TVC, and ΔE under zero- and first-order reactions, relative errors between experimental and predicted data were calculated and are presented in Table 4, using day 15 of storage as an example. The shelf life prediction model under first-order reaction (Equation (7b)) could predict storage changes of TVC with the lowest average relative error (14.79%). For the shelf life prediction models under zero-order reactions, the average relative errors between experimental and predicted values of hardness, TVC, and ΔE all exceeded 15%. Additionally, the average relative errors of the shelf life prediction models based on ΔE were the largest (>30%). Therefore, the Arrhenius model under first-order reaction based on TVC (Equation (7b)) was the best-fitted model for modeling the shelf life of HPP marinated lotus root slices during storage at 4–45 °C.

## 3. Materials and Methods

### 3.1. Materials and Chemicals

Fresh lotus roots (*Nelumbo nucifera* Gaertn.) segments with an average diameter of 60 mm were provided by Jiangsu Jinsha Foods Co., Ltd. (Xinghua, China). The concentrated spice brine (pH = 4.37, total soluble solids = 16.0° Brix) was supplied by Jiangsu Teweinong Biological Technology Development Co., Ltd. (Xinghua, China). Cyclohexanone (gas chromatography grade) was purchased from ANPEL Laboratory Technologies, Inc. (Shanghai, China). *N*-Alkane (C_7_-C_30_) standards for qualitative analysis were purchased from Sigma (St. Louis, MO, USA). Plate count agar, NaCl, and other analytical-grade reagents were purchased from Beijing Solarbio Science & Technology Co., Ltd., Beijing, China.

### 3.2. Preparation of Marinated Lotus Root Slices

Lotus roots were cleaned, peeled, sliced into 7 mm thick disks, and blanched with boiling water for 5 min (1:4, *w/v*). The lotus root slices were cooled in icy water for 10 min and set as a blank group, followed by TS or HPP treatment. According to the instructions for the concentrated spice brine provided by the manufacturer, water and concentrated brine (4:1, *w/w*) were added to a crock and boiled. The dreg-removed brine was used to marinate the lotus root slices.

Samples (100.0 ± 0.1 g) for TS treatment were boiled in boiling brine (100 °C, 100.0 g) for 20 min, soaked for 5 h in the brine without heating (25 °C), and vacuum-packed without brine using a vacuum-packing machine (Deli Group Co., Ltd., Ningbo, China) in a clear polyethylene retort pouch (15 cm × 22 cm).

Samples (100.0 ± 0.1 g) for HPP treatment were vacuum-packed into a retort pouch containing 15 g of brine, subjected to a high hydrostatic pressure pressurization unit (CQC30L-600, Suyuanzhongtian Scientific Co., Ltd., Beijing, China), and treated at 550 MPa for 20 min at room temperature (approximately 25 °C). Distilled water was used as the pressure-transmitting fluid, and the pressurization rate was approximately 120 MPa/min. Decompression (<3 s) was performed immediately after the treatment to minimize adiabatic heating. The treatment time did not include the pressure increase or release time.

Lotus root slices (100.0 ± 0.1 g) vacuum packed with 15 g of brine were boiled at 100 °C for 20 min and left at 25 °C for 5 h and set as the control group.

### 3.3. Quality Evaluation

#### 3.3.1. Analysis of Volatile Compounds

- Extraction of volatile compounds

The extraction of volatile compounds was performed following the method described by Shen et al. [59] using solid-phase microextraction (SPME), with minor modifications. Chopped samples (2.5 g) were homogenized and transferred into a headspace bottle (20 mL, ANPEL Laboratory Technologies Inc., Shanghai, China) containing 50 μL of 1000 times diluted cyclohexanone as an internal standard. The bottles were sealed using a PTFE-silicone septum and equilibrated at 50 °C for 15 min with agitation. Next, a 50/30 μm divinylbenzene/carboxen/polydimethylsiloxane (DVB/CAR/PDMS) SPME fiber (Supelco, Bellefonte, PA, USA) was exposed to the headspace of the samples for 40 min at the same temperature without stirring. Finally, the fiber was withdrawn and introduced into the GC injector at 250 °C for 5 min.

- Gas chromatography–mass spectrometry (GC-MS) analysis

GC-MS measurements were conducted following the method of previous studies [60], with minor modifications, using an Agilent 7890 gas chromatography system (Agilent Technologies, Santa Clara, CA, USA) equipped with an Agilent 5975C series mass spectrometer. The volatile compounds were isolated with a DB-WAX (30 m × 320 μm i.d. × 0.25 μm) fused silica capillary column (Agilent Technologies). Helium (purity ≥ 99.999%) was used as the carrier gas at a rate of 1.0 mL/min constant flow. The oven temperature was maintained at 40 °C for 12 min, increased at a rate of 3 °C/min to 108 °C, and then maintained at 108 °C for 2 min, followed by an increase to 250 °C at a rate of 5 °C/min for 5 min. Mass spectrometry was performed in the electron impact mode of 70 eV with a scan range of 450–550 m/z.

- Identification and quantification analysis

The volatile compounds in HPP and TS marinated lotus root slices were identified by comparing sample mass spectra with those of the standard NIST12 database and by comparing the calculated linear retention indices (LRIs) with the open-access data of the NIST WebBook. The LRIs of volatile compounds were calculated using the retention times (RTs) of a liquid injection of 1 μL of C_7_-C_30_ *n*-alkane standards obtained using the same GC-MS temperature program. A difference value below 20 between the calculated LRI values and those from the NIST Chemistry WebBook (https://webbook.nist.gov/chemistry/ (accessed on 31 October 2021)) was considered acceptable. LRI was calculated using the following equation:

(9) $$\begin{array}{c} \mathrm{LRI}=100N+\frac{100n\left(t_{\mathrm{Ra}}-t_{\mathrm{RN}}\right)}{t_{R\left(N+n\right)}-t_{\mathrm{RN}}} \end{array}$$

where N is the number of carbon atoms of *n*-alkanes immediately before the RT of the compound, n is the difference in the number of carbon atoms of *n*-alkanes immediately before and after the RT of the compound, t_Ra_ is the RT of the compound, t_RN_ is the RT of *n*-alkanes immediately before the compound, and t_R(N+n)_ is the RT of *n*-alkanes immediately after the compound.

Quantification of volatile compounds in HPP and TS marinated lotus root slices was performed using cyclohexanone as an internal standard. The peak areas were normalized to the cyclohexanone added to each sample. The concentrations of the identified compounds were calculated from the ratio of the peak area to that of the internal standard.

#### 3.3.2. Determination of pH and Hardness

Marinated lotus root slices were removed from the pouch and homogenized (FSH-2A, Fangke Instrument Co., Ltd., Changzhou, China). The pH of the mixture was measured at 25 °C using a Crison basic 20 pH meter (Crison, Spain).

Texture is an important quality indicator of marinated vegetables, and in this study, hardness was chosen as an indicator of the texture parameter [2,61]. Hardness measurements were performed within 1 h after HPP or TS treatment using a TAXT plus texture analyzer (Stable Micro Systems, Surrey, England) as described by Dong et al. [62], with some modifications. The compression force at 30% strain was obtained using a cylindrical flat probe (50 mm diameter, aluminum). The sample was cut into cubes (1.0 cm × 1.0 cm × 0.7 cm); placed on the platform with the square side face up; and measured with a 5 g trigger at 1, 1, and 5 mm/s of pre-speed, test speed, and post-speed, respectively. The hardness of each sample was defined as the peak force at a strain of 30%.

#### 3.3.3. Measurements of Color

The color of the marinated lotus root slices was measured at room temperature (approximately 25 °C) using a color difference meter (ColorQuest XE, Hunter Associated Laboratory Inc., USA) in the reflectance mode immediately after opening the pouches. The light source was set to D65 with a 0.375-inch observation aperture and a 10° observation angle. The chromometer was calibrated using a white standard before the samples were measured. The color was recorded in units of L*, a*, and b* uniform color spaces. L* indicates lightness, the a* scale ranges from negative values for green to positive values for red, and the b* scale ranges from negative values for blue to positive values for yellow. The total color difference (ΔE) was calculated using the following equation:

(10) $$\begin{array}{c} \Delta E=\sqrt[{2}]{{\left(L^{*}-L^{*}{}_{0}\right)}^{2}+{\left(a^{*}-a^{*}{}_{0}\right)}^{2}+{\left(b^{*}-b^{*}{}_{0}\right)}^{2}} \end{array}$$

where L*_0_, a*_0_, and b*_0_ are the values for the blank lotus root slices.

#### 3.3.4. Determination of TVC

The number of microorganisms in the marinated lotus root slices was determined using the total plate counting method. Marinated lotus root slices (10.0 g) were chopped and homogenized with 90.0 mL sterile 0.85% NaCl solution; the sample was then 10-fold serially diluted with sterile 0.85% NaCl solution, and 1.0 mL of each dilution was plated onto a nutrient agar plate (20.0 mL). The nutrient agar was used for detecting TVC after incubation at 37 °C for 48 h. The microorganism numbers of the samples were enumerated as log_10_ of CFU/g.

### 3.4. Storage Conditions

The packaged HPP (including 100.0 g of lotus root slices and 15.0 g of brine) and TS (including 100.0 g of lotus root slices without brine) marinated lotus root slices were stored at 4, 25, and 45 °C in an incubator (PHX, Ningbo Laifu Technology Co., Ltd., Ningbo, China) until the sample microbial load exceeded the usually accepted limit of 5 log_10_ CFU/g [63]. Samples were analyzed on days 0, 6, 15, 25, 35, 45, 55, 70, and 80 at 4 °C; 0, 4, 9, 15, 25, 35, 45, and 55 at 25 °C; and 0, 2, 4, 6, 9, 12, 15, and 18 at 45 °C to determine chemical and microbiological quality changes during storage. Each time, three pouches of marinated lotus root slices from each batch were collected for duplicate measurements.

### 3.5. Statistics Analysis

All results are presented as the average ± standard deviation (SD). One-way analysis of variance (ANOVA) and Duncan’s multiple range test were conducted to determine significant differences between samples using SPSS (version 25.0; Chicago, IL, USA), where the significance level was set at *p* < 0.05. GraphPad Prism 8.0 (San Diego, CA, USA) and Origin 2019 software (Northampton, MA, USA) were used for data plotting.

## 4. Conclusions

In this study, HPP demonstrated the potential to improve marinated food quality while resulting in brine savings of approximately 85%. Both HPP (550 MPa, 20 min) and traditional TS (100 °C, 20 min; 25 °C, 5 h) marinated lotus root slices showed a decrease in TVC on day 0 and slight variations in the visual appeal, texture, and aroma qualities. During storage at different temperatures, HPP and TS samples exhibited similar microbiological safety. At the end of the storage period, less deterioration in hardness and color was observed in HPP marinated lotus root slices than in TS samples. The Arrhenius model under first-order reaction based on TVC was the best-fitted model for modeling the shelf life of HPP marinated lotus root slices during storage at 4–45 °C. Therefore, HPP can be an alternative and efficient option to extend the shelf life of marinated lotus root slices with less effect on the quality properties and less brine waste.

## Figures and Tables

**Table 1:** Volatile profiles of HPP and TS marinated lotus root slices.

No.	Compounds	Aroma Descriptors ^1^	LRI	Concentration (μg/kg) ^2^		
				Blank	HPP	TS
	aldehydes					
A1	1-Nonanal	Waxy, rose	1393	12.38 ± 2.17 b	247.32 ± 97.69 b	695.10 ± 295.09 a
A2	Benzaldehyde	Sharp, sweet	1510	n.d.	776.66 ± 173.01 b	1209.04 ± 312.11 a
A3	Phenylacetaldehyde	Green, sweet	1629	n.d.	497.25 ± 330.72 ab	906.15 ± 5.27 a
A4	2-Phenyl-2-butenal	Sweet, narcissus	1919	n.d.	47.75 ± 5.37 a	n.d.
A5	Cinnamaldehyde	Sweet, spicy	2030	n.d.	14.07 ± 4.38 a	n.d.
A6	Cocal	Bitter, cocoa	2069	n.d.	25.78 ± 6.45 a	n.d.
	Subtotal			12.38 ± 2.17 c	1608.83 ± 617.62 b	2810.29 ± 612.47 a
	heterocyclic compounds					
B1	Furfural	Sweet, woody	1457	15.18 ± 9.63 c	787.57 ± 118.23 b	1051.82 ± 98.78 a
B2	5-Methyl furfural	Spice, caramel	1565	n.d.	158.65 ± 39.21 b	375.96 ± 38.36 a
B3	Acetylpyrazine	Popcorn, nutty	1614	n.d.	175.88 ± 62.5 b	390.92 ± 36.98 a
B4	Furfuryl alcohol	Alcoholic, chemical	1658	n.d.	321.83 ± 23.09 a	n.d.
B5	2-Acetyl pyrrole	Musty, nut	1958	n.d.	332.81 ± 45.26 b	579.91 ± 44.95 a
B6	Ethyl maltol	Sweet, caramel	1998	n.d.	3885.19 ± 860.53 a	4640.65 ± 963.37 a
B7	5-(2-Hydroxyethyl)-4-methylthiazole	Fatty, cooked beef	2292	n.d.	95.44 ± 18.88 a	94.01 ± 17.08 a
	Subtotal			15.18 ± 9.63 c	5757.37 ± 1167.70 b	7133.27 ± 1199.52 a
	alcohols					
C1	Benzyl alcohol	Floral, rose	1865	n.d.	2141.2 ± 265.17 b	2785.97 ± 244.55 a
C2	Phenethyl alcohol	Floral, rose	1898	n.d.	740.32 ± 96.60 a	843.44 ± 75.53 a
	Subtotal			n.d.	2881.52 ± 361.77 b	3629.41 ± 320.08 a
	ethers					
D1	Di-n-decyl ether		1199	0.40 ± 0.11 a	n.d.	n.d.
D2	4-Allylanisole	Sweet, sassafras	1662	n.d.	235.65 ± 20.07 a	252.35 ± 23.80 a
D3	cis-Anethol	Sweet, anise	1818	n.d.	1573.04 ± 240.16 a	1725.51 ± 183.42 a
D4	Methyl eugenol	Sweet, fresh	2010	n.d.	488.64 ± 115.99 a	508.58 ± 41.26 a
D5	Methyl isoeugenol	Spicy, clove	2179	n.d.	72.13 ± 12.56 a	61.29 ± 25.40 a
D6	Elemicin	Spice, flower	2224	n.d.	202.00 ± 53.42 a	223.15 ± 16.60 a
D7	Myristicin	Spicy, warm	2252	n.d.	307.76 ± 85.43 b	571.90 ± 38.19 a
	Subtotal			0.40 ± 0.11 c	2879.22 ± 527.63 b	3342.78 ± 328.67 a
	esters					
E1	Methoxyacetic acid, 2-tridecyl ester		1309	2.58 ± 0.69 a	n.d.	n.d.
E2	Ethyl caprylate	Fruity, wine	1432	n.d.	122.46 ± 21.05 b	291.75 ± 110.42 a
E3	Diethyl succinate	Mild, fruity	1678	n.d.	233.68 ± 24.37 b	334.37 ± 22.89 a
E4	Ethyl myristate	Sweet, waxy	2055	n.d.	n.d.	206.98 ± 34.35 a
E5	Isopropyl myristate	Oily, fatty	2074	8.11 ± 1.16 a	n.d.	n.d.
E6	Ethyl palmitate	Mild, waxy	2263	n.d.	53.96 ± 10.99 b	202.63 ± 53.48 a
E7	2-Ethylhexyl salicylate	Mild, orchid	2314	7.74 ± 2.05 a	n.d.	n.d.
E8	Dibutyl phthalate	Faint	2698	6.72 ± 2.96 a	n.d.	n.d.
	Subtotal			25.15 ± 6.86 c	410.10 ± 56.41 b	1035.73 ± 221.14 a
	phenols					
F1	Butylated hydroxytoluene	Phenolic, camphor	1912	n.d.	63.31 ± 9.43 a	n.d.
F2	Phenol	Phenolic, plastic	1994	1.56 ± 0.34 c	44.56 ± 4.15 b	271.53 ± 32.80 a
F3	4-Ethyl-2-methoxyphenol	Spicy, smoky	2019	n.d.	156.47 ± 35.34 a	n.d.
F4	Eugenol	Sweet, spicy	2156	n.d.	9546.86 ± 2096.10 a	9660.94 ± 831.41 a
F5	4-Hydroxy-3-methoxystyrene	Sweet, spicy	2185	n.d.	48.61 ± 7.23 a	n.d.
F6	Isoeugenol	Sweet, spicy	2245	n.d.	166.14 ± 39.81 a	76.34 ± 20.03 b
F7	2,4-Di-tert-butylphenol	Phenolic	2333	19.39 ± 2.73 c	494.25 ± 56.39 b	946.03 ± 229.91 a
	Subtotal			20.95 ± 3.07 b	10,520.20 ± 2248.45 a	10,954.84 ± 1114.15 a
	alkyls					
G1	Hexane		1017	16.17 ± 2.29 a	n.d.	n.d.
G2	Undecane		1081	16.98 ± 2.36 a	n.d.	n.d.
G3	Dodecane		1166	6.84 ± 2.07 a	n.d.	n.d.
G4	Hexadecane		1641	6.22 ± 0.81 a	n.d.	n.d.
G5	Eicosane	Waxy	1636	7.00 ± 4.37 a	n.d.	n.d.
	Subtotal			53.21 ± 11.90 a	n.d.	n.d.
	others					
H1	1-Methylethyl-benzene		1138	15.96 ± 6.02 a	n.d.	n.d.
H2	DL-Limonene	Citrus, herbal	1173	3.36 ± 0.12 a	4.91 ± 0.22 a	4.94 ± 6.11 a
H3	6-Methyl-5-hepten-2-one	Citrus, green	1329	3.40 ± 1.29 a	n.d.	n.d.
H4	1,2,3-Trimethyl-4-[(*E*)-prop-1-enyl]naphthalene		2288	0.76 ± 0.02 a	n.d.	n.d.
	Subtotal			23.48 ± 7.45 a	4.91 ± 0.22 b	4.94 ± 6.11 b
	Total			150.75 ± 41.19 b	24,062.15 ± 4979.80 a	28,911.26 ± 3802.14 a

^1^ Reference aroma descriptors from the LRI & Odor Database (http://www.odour.org.uk/, accessed on 14 September 2022). ^2^ Values are given as mean ± standard deviation (SD; n = 3). n.d., not detected. Different lowercase letters in the same row indicate significant differences at *p* < 0.05. LRI, linear retention index; HPP, high-pressure processing; TS, thermal-and-soaking.

**Table 2:** Quality properties of HPP and TS marinated lotus root slices.

		Blank	HPP	TS
TVC (log_10_ CFU/g)		3.98 ± 0.04 a	1.68 ± 0.02 b	1.60 ± 0.16 b
Hardness (g)		14,208.14 ± 1985.80 a	14,820.45 ± 814.85 a	11,955.97 ± 1040.45 b
pH		6.47 ± 0.02 a	4.82 ± 0.03 b	4.73 ± 0.04 c
Color	L*	76.35 ± 1.42 a	51.74 ± 1.52 b	48.36 ± 0.68 c
	a*	−0.88 ± 0.05 c	7.58 ± 0.57 b	8.48 ± 0.43 a
	b*	2.65 ± 0.32 c	21.56 ± 1.20 b	23.95 ± 0.74 a
	ΔE	-	32.20 ± 0.45 b	36.41 ± 0.39 a

Different lowercase letters in the same row indicate significant differences among the treatments (*p* < 0.05). HPP, high-pressure processing; TS, thermal-and-soaking; TVC, total viable count; ΔE, total color difference.

**Table 3:** Changes in total color difference of HPP and TS marinated lotus root slices during storage.

Storage Temperature (°C)	Storage Time (Days)	HPP	TS
45	0	32.20 ± 0.45 j	36.41 ± 0.39 gh
	2	35.34 ± 0.92 hi	38.08 ± 0.44 cde
	4	35.37 ± 0.31 hi	37.84 ± 0.11 def
	6	35.18 ± 0.75 i	36.76 ± 1.45 fg
	9	35.33 ± 0.18 hi	37.12 ± 0.50 efg
	12	37.07 ± 1.14 efg	39.76 ± 1.68 b
	15	38.63 ± 0.20 bcd	41.38 ± 0.90 a
	18	39.11 ± 0.29 bc	42.07 ± 0.29 a
25	0	32.20 ± 0.45 k	36.41 ± 0.39 fgh
	4	33.37 ± 1.90 jk	36.13 ± 0.72 fghi
	9	34.40 ± 0.89 ij	37.52 ± 0.71 efgh
	15	35.65 ± 0.73 hi	38.01 ± 0.32 def
	25	35.88 ± 0.25 ghi	38.69 ± 1.68 de
	35	35.76 ± 0.93 ghi	41.85 ± 0.50 ab
	45	37.69 ± 1.34 efg	40.88 ± 1.61 bc
	55	39.60 ± 0.46 cd	42.92 ± 1.57 a
4	0	32.20 ± 0.45 k	36.41 ± 0.39 efg
	6	33.08 ± 1.07 jk	35.28 ± 0.45 ghi
	15	33.87 ± 0.84 ijk	37.66 ± 0.41 def
	25	34.44 ± 0.79 hij	38.02 ± 0.15 cde
	35	34.49 ± 2.21 ghij	39.88 ± 0.62 bc
	45	35.01 ± 1.59 ghi	39.82 ± 0.19 bc
	55	36.08 ± 2.44 fgh	40.29 ± 1.56 b
	70	38.60 ± 1.84 bcd	42.71 ± 1.36 a
	80	40.19 ± 0.25 b	42.81 ± 0.38 a

Data are expressed as the mean standard deviation (n = 4). Different lowercase letters in the same column for each storage temperature indicate significant differences at *p* < 0.05. HPP, high-pressure processing; TS, thermal-and-soaking.

**Table 4:** Activation energy (Ea) and frequency coefficient (k_0_) for the zero- and first-order change of quality indices; relative errors between experimental and predicted shelf life for HPP marinated lotus root slices at day 15.

		k_0_	Ea (kJ/mol)	Relative Error ^1^			
				Stored at 4 °C	Stored at 25 °C	Stored at 45 °C	Average
Hardness	Zero-order	4.50 × 10^6^	25.00	38.90%	3.98%	6.25%	16.37%
	First-order	5.57 × 10^2^	25.33	31.03%	15.26%	1.56%	15.95%
TVC	Zero-order	6.49 × 10^3^	27.92	15.21%	20.13%	16.06%	17.14%
	First-order	1.79 × 10^3^	27.47	16.02%	8.07%	20.27%	14.79%
ΔE	Zero-order	1.31 × 10^3^	22.44	56.30%	18.11%	29.44%	34.62%
	First-order	32.54	22.15	56.83%	21.20%	29.12%	35.71%

^1^ Relative error = (|Predicted shelf life (day) − 15|/15) × 100%. HPP, high-pressure processing; TS, thermal-and-soaking; TVC, total viable count; ΔE, total color difference.

## Data Availability

Data is contained within the article or Supplementary Material.

## Supplementary Materials

The following supporting information can be downloaded at: https://www.mdpi.com/article/10.3390/molecules27196506/s1, Table S1: Changes in color of HPP and TS marinated lotus root slices during storage. Table S2: Reaction rate constant k and determination coefficient R^2^ for zero- and first-order regression of HPP-treated marinated lotus root slices at different temperatures.
