# Supplementary material for: Production of Marinated Chinese Lotus Root Slices Using High-Pressure Processing as an Alternative to Traditional Thermal-and-Soaking Procedure
Source: Molecules. 2022 Oct 2;27(19):6506. doi: 10.3390/molecules27196506 (PMC9571789; doi:10.3390/molecules27196506)
Supplement: Supplementary file 1 [file molecules-27-06506-s001.zip › molecules-1904368-supplementary.pdf]

Table S1. Changes in color of HPP and TS marinated lotus root slices during storage

Storage temperature (°C)	Storage time (days)	HPP			TS		
		L*	a*	b*	L*	a*	b*
Blank	0	76.35±1.42	-0.88±0.05	2.65±0.32			
45	0	51.74±1.52b	7.58±0.57g	21.56±1.20bc	48.36±0.68c	8.48±0.43def	23.95±0.74a
	2	47.31±1.03cd	7.77±0.80fg	20.82±0.31cd	45.01±0.75e	8.71±0.20d	22.03±0.88b
	4	46.99±0.38d	7.94±0.05efg	20.29±0.02de	44.52±0.39e	8.60±0.43de	20.79±0.26cd
	6	46.63±0.31d	7.88±0.11efg	19.29±1.05ef	44.42±1.17e	8.82±0.33d	18.05±1.19gh
	9	46.28±0.15d	8.63±0.72de	18.56±0.26fg	44.29±0.55e	9.98±0.44b	17.89±0.50gh
	12	44.24±1.21e	9.08±0.46cd	18.24±0.69fgh	41.26±1.59f	10.08±0.78ab	17.77±0.77gh
	15	42.19±0.08f	8.38±0.21def	18.12±0.31fgh	39.27±0.68g	10.13±0.26ab	17.33±1.37h
	18	41.64±0.23f	9.64±0.42bc	17.27±0.21h	38.57±0.01g	10.72±0.33a	17.05±0.77h
25	0	51.74±1.52b	7.58±0.57e	21.56±1.20bc	48.36±0.68de	8.48±0.43cde	23.95±0.74a
	4	50.60±2.80bc	8.20±1.18cde	21.73±1.29bc	48.20±0.68de	8.55±0.30cde	23.22±1.10a
	9	48.85±1.00cd	7.80±0.37de	21.39±0.29bcd	46.67±0.98def	8.54±0.37cde	23.55±0.47a
	15	47.02±0.77def	8.32±0.16cde	20.70±0.29bcde	44.84±0.23fg	9.14±0.25c	21.38±1.00bcd
	25	46.69±0.07def	8.75±1.00cd	20.36±0.24cdef	42.89±2.50gh	7.69±0.37de	20.01±0.94defg
	35	46.06±0.93ef	8.42±1.34cde	19.19±1.09fgh	41.01±0.89hi	10.62±0.37b	21.85±1.23b
	45	43.77±2.04g	8.63±0.88cde	18.95±1.04gh	40.63±1.49hi	11.23±0.48ab	18.36±1.21h
	55	41.23±0.10h	8.97±0.56c	18.02±1.41h	38.88±1.42i	11.71±0.45a	19.35±0.99efgh
4	0	51.74±1.52b	7.58±0.57g	21.56±1.20bcd	48.36±0.68cde	8.48±0.43fg	23.95±0.74a
	6	50.46±1.43bc	7.46±0.80g	21.42±1.23bcd	48.23±0.30cde	8.76±0.15ef	21.63±0.52bcd
	15	49.13±0.64cd	8.32±1.10fg	20.56±0.96cde	46.40±0.45efg	9.97±0.23cd	22.70±0.79ab
	25	48.29±0.82cde	8.33±0.17fg	20.33±1.19def	45.63±0.51fgh	9.95±0.33cd	22.23±1.20bc
	35	48.46±4.21cde	8.80±0.41ef	20.18±2.70def	44.72±1.02ghi	11.00±1.26ab	23.80±0.96a
	45	47.27±1.80def	8.47±0.28fg	19.69±0.63efg	43.73±0.90hij	11.02±0.58ab	22.07±0.75bc
	55	45.94±3.28efgh	9.17±0.02def	19.19±0.95efg	41.89±2.02jk	9.65±0.64de	20.63±0.81cde
	70	42.82±2.23ijk	10.07±0.69bcd	18.29±0.80gh	38.49±2.16l	11.20±0.81a	18.22±1.24gh
	80	41.21±0.87k	10.87±0.64abc	18.17±1.20gh	38.00±0.54l	11.02±0.12ab	17.50±0.19h

Data are expressed as the mean standard deviation (n = 4). Different lowercase letters in the same column for each storage temperature indicate significant differences at $p < 0.05$. HPP, high pressure processing; TS, thermal-and-soaking.

Table S2. Reaction rate constant k and determination coefficient R^2 for zero- and first- order regression of HPP-treated marinated lotus root slices at different temperatures

Index	Storage temperature (°C)	Zero-order regression			$\overline{R}^2$	First-order regression			$\overline{R}^2$
		k	C_0	R^2		k	C_0	R^2	
Hardness	4	-102.27	13411	0.9779	0.9524	-0.011	13933	0.9939	0.9688
	25	-129.85	13013	0.9497		-0.014	13352	0.9620	
	45	-428.78	13224	0.9295		-0.047	13683	0.9504	
TVC	4	0.0407	1.3716	0.9475	0.9571	0.0138	1.6117	0.9912	0.9900
	25	0.0601	1.6488	0.9837		0.0195	1.8091	0.9862	
	45	0.2002	1.2536	0.9401		0.0663	1.5485	0.9925	
ΔE	4	0.0887	32.066	0.9258	0.9000	0.0025	32.189	0.9367	0.9029
	25	0.1118	32.941	0.9122		0.0031	32.992	0.9117	
	45	0.3208	33.382	0.8610		0.0089	33.421	0.8603	
